# Gender-Specificity of Initial and Controlled Visual Attention to Sexual Stimuli in Androphilic Women and Gynephilic Men

**DOI:** 10.1371/journal.pone.0152785

**Published:** 2016-04-18

**Authors:** Samantha J. Dawson, Meredith L. Chivers

**Affiliations:** Department of Psychology, Queen’s University, Kingston, Ontario, Canada; Knox College, UNITED STATES

## Abstract

Research across groups and methods consistently finds a gender difference in patterns of specificity of genital response; however, empirically supported mechanisms to explain this difference are lacking. The information-processing model of sexual arousal posits that automatic and controlled cognitive processes are requisite for the generation of sexual responses. Androphilic women’s gender-nonspecific response patterns may be the result of sexually-relevant cues that are common to both preferred and nonpreferred genders capturing attention and initiating an automatic sexual response, whereas men’s attentional system may be biased towards the detection and response to sexually-preferred cues only. In the present study, we used eye tracking to assess visual attention to sexually-preferred and nonpreferred cues in a sample of androphilic women and gynephilic men. Results support predictions from the information-processing model regarding gendered processing of sexual stimuli in men and women. Men’s initial attention patterns were gender-specific, whereas women’s were nonspecific. In contrast, both men and women exhibited gender-specific patterns of controlled attention, although this effect was stronger among men. Finally, measures of attention and self-reported attraction were positively related in both men and women. These findings are discussed in the context of the information-processing model and evolutionary mechanisms that may have evolved to promote gendered attentional systems.

## Introduction

Sexual response is an emotional state emerging from interactions among physiological responses (e.g., genital vasocongestion), cognitive processing of sexual cues (e.g., attention), and affective responses [1, 2]. Gender differences in the stimulus cues that elicit sexual response are well established. Men’s genital and self-reported sexual responses are category-specific, readily distinguishing between preferred and nonpreferred sexual stimuli, thereby exhibiting a bias towards cues or features that correspond with their stated sexual attractions [3–12]. In contrast, androphilic (i.e., sexually attracted to men) women’s genital response patterns, and sometimes self-reported arousal patterns, are nonspecific because they typically do not distinguish between preferred and nonpreferred sexual stimuli and therefore show no bias towards stimulus cues or features consistent with their stated sexual attractions (i.e., category-nonspecific) [4, 5, 8, 11, 13–17].

Empirically supported mechanisms to explain the observed gender difference in the specificity of genital sexual responses are currently lacking. The information-processing model (IPM) of sexual response posits that attention to sexual cues initiates sexual responding [18], thus patterns of activation and attention may differ between men and women and may contribute to the differences observed in genital response to sexual stimuli. According to the IPM, two cognitive processes are involved when generating a sexual response. *Automatic processing* of sexual stimuli involves the pre-attentive, unconscious detection of sexually-relevant features. These features, if matched with representations of sexual stimuli in a person’s implicit memory, will activate a genital response [18–21]. *Controlled attentional processing* involves the integration of automatic processes, conscious awareness, memory, and the corresponding sexual response. As the sexual response develops, sexual aspects of the stimulus capture focal attention and activate contents in a person’s explicit memory, allowing the sexual meanings inherent in the stimuli to be elaborated. This elaboration facilitates sexual responding, including the conscious awareness of sexual arousal. Studies manipulating the degree of absorption or elaboration in sexual stimuli have found that increased absorption leads to the magnification of sexual response [22–25], whereas the inhibition of elaborative processing of sexual cues via distraction weakens sexual responding [2, 22, 26–29].

An IPM-derived hypothesis for the gender difference in specificity of genital sexual response might predict that androphilic women’s gender-nonspecific pattern is the result of sexually-relevant cues present in both preferred and nonpreferred sexual targets (e.g., genitals, chest/breasts, body shape) capturing attention and initiating a sexual response. In contrast, the IPM would predict that men’s attentional system is biased towards the detection and elaboration of sexually-preferred cues only (e.g., cues present in female targets—breasts, vulva). Recently, Huberman, Maracle, and Chivers [30] found initial support for this hypothesis. They observed that self-reported attention to sexual cues was a significant mediator of gender-specific (i.e., greater arousal to one’s preferred gender) genital and self-reported sexual arousal in men, but not women. Although supportive of the IPM, self-report measures of attention do not provide sufficiently detailed data to explore the relationship between automatic and conscious attentional processes and sexual responding as proposed by the IPM.

### Measures of Attention

Eyetracking is a direct way to assess stimulus processing through the acquisition of detailed data on visual fixations—the length of time during which the eye does not move and information is acquired and processed [31]. Fixations, then, are a direct measure of visual attention and can be used to assess visual processing of complex stimuli. Moreover, eyetracking can identify which aspects or regions of stimuli initially capture attention [32]. Relevant to the IPM, eyetracking can provide data regarding the time course of visual attention, so it is possible to examine both initial attention (i.e., covert automatic allocation of attentional resources) and controlled attention (i.e., overt orienting of gaze and gaze duration) [33].

Controlled shifts in gaze are preceded by initial shifts in attention allocation [33, 34]. There are two main indices of initial attentional processing using eyetracking. The first involves recording the time to first fixation, or the length of time (in seconds) for an overt or conscious shift in visual attention to a particular stimulus region after the covert or automatic shift in attention is elicited. Validation of time to first fixation as a measure of initial attentional engagement with emotional or evolutionarily relevant information is demonstrated in studies where people take significantly less time to fixate on emotional faces (e.g., happy, sad, threatening) than faces with a neutral expression [32, 35, 36], as well as significantly less time to fixate on fearful or threatening stimuli compared to non-threatening stimuli [37, 38]. The second index of initial attention involves examining the number of first fixations landing on a region of interest. Again, the prediction is that regions that capture attention and motivate overt shifts in attention will do so at a greater frequency than regions that do not capture attention [32, 36, 37–41]. Similar to studies assessing time to first fixation, emotional faces or threatening stimuli yield significantly higher probabilities or frequencies of first fixations than do neutral faces or nonthreatening stimuli [32, 36–39].

Indices of consciously controlled attentional processes include total fixation duration (total amount of time spent looking at a particular region) and total fixation count (total number of fixations to a particular region) [42, 43]. These measures have been used in affective neuroscience to detect consciously controlled biases to faces differing in emotional valence. For example, affectively-valenced stimuli (e.g., happy, angry, sad, threatening) capture attention for significantly longer durations than do affectively-neutral stimuli [32, 36–39, 42, 43].

### Assessing Attention to Sexual Cues

Gender-specific sexual responding in men and gender-nonspecific responding in women may be associated with initial and controlled attentional biases to sexual cues [40, 44–48]. Using erotic images of heterosexual couples as stimuli, gynephilic (i.e., sexually attracted to women) men show significantly more fixations and longer fixation durations to the female target, whereas for women, both the female and the male targets attract and sustain visual attention [45–47]. Though compelling, it is possible that the stimuli and paradigms used in the majority of eye-tracking studies have influenced the patterns of results observed [45–47]. Images of couples contain additional cues such as the setting or sexual acts that may attract and sustain attention independent of the persons depicted. Along these lines, Rupp and Wallen [47] reported a gender difference in the degree to which contextual cues (e.g., background, clothing) attracted visual attention. They observed that these contextual cues attracted women’s attention significantly more than men’s. Although not examined in their study, Lykins et al. [45] noted that women may have engaged in self-other comparisons during the presentation of images of couples, thus contributing to women’s attention on the female target in the image.

To counter these types of limitations, a *forced attention paradigm* has been used to examine attentional biases to stimuli that differ in affective value. Rather than presenting one image with multiple targets and affective meanings, two distinct targets that differ in the variable of interest (e.g., emotion) [32] are presented simultaneously, allowing for biases in attention to be attributed to differences in the variable of interest. Using a free-viewing version of this task, Bradley, Costa, and Lang [49] presented women and men with two images simultaneously (i.e., a neutral image and either an image of a nude male or a nude female). Consistent with studies using images of couples, men exhibited a gender-specific pattern of visual attention throughout the stimulus presentation, whereas women exhibited a gender-nonspecific pattern of visual attention.

Recently, Fromberger et al. [40] used a modified version of the forced attention paradigm to examine initial and controlled attentional processes as they relate to sexual attractions. Fromberger et al. examined gynephilic men’s visual attention biases to preferred and nonpreferred sexual stimuli based on gender preferences. They found that men showed attentional biases towards images of adult women—their preferred sexual target. Consistent with predictions from the IPM, men were significantly more likely to first look at their sexually-preferred target (initial attentional processing), preferred targets captured their attention for significantly longer than sexually nonpreferred targets (controlled attentional processing), and men reported higher ratings of sexual attraction towards sexually-preferred targets. More recently, this research group has examined visual attention biases related to age preferences among men with teleiophilic (i.e., sexual attraction to adults) and pedophilic (i.e., sexual attraction to children) attractions. Similar to the findings documenting attentional biases towards stimuli based on gender preferences, they observed that both groups of men exhibited attentional biases towards the detection of stimuli that matched their sexual preference in terms of the age of the targets depicted [50, 51]. The three Fromberger et al. studies described have only included men, so it is unknown whether women would show similar attentional biases, or nonspecific attention patterns when simultaneously presented with preferred and nonpreferred targets, similar to studies showing gender-nonspecific sexual responding in androphilic women.

### The Current Study

In the current study, we tested predictions from the IPM with respect to patterns of initial and controlled attention towards preferred and nonpreferred sexual targets in androphilic women and gynephilic men. In light of the findings from the few studies of visual attention to preferred and nonpreferred sexual stimuli, we predicted a gender difference in patterns of attention towards sexually-preferred and nonpreferred targets for both initial and controlled attention measures, such that men would exhibit an attentional bias towards their preferred target, whereas women would not. Of note, the gender difference examined in the current study is limited to the comparison of gynephilic men and androphilic women, and as such cannot be interpreted as a comprehensive gender difference that transcends sexual orientation. Specifically, we predicted that men would i) initially orient more quickly (time to first fixation) and ii) orient more often (number of first fixations) towards preferred sexual targets (i.e., female images). We also expected that iii) sexually-preferred targets would capture men’s attention for significantly longer (total fixation duration) and iv) more frequently (total fixation count) than nonpreferred targets. We expected gender-nonspecific patterns of initial and controlled attention in women, specifically predicting that women would v) orient similarly quickly to sexually-preferred and nonpreferred targets, and vi) similarly often towards sexually-preferred and nonpreferred targets. We also expected that vii) sexually-preferred and nonpreferred targets would capture women’s attention for similar amounts of time and viii) at similar frequencies. Given that attention can be captured and held for many reasons, we also examined patterns of self-reported attraction to the male and female targets in order to investigate correlates of attention. We expected gaze times and attraction ratings to be positively related for men and women.

## Materials and Methods

### Participants

Participants were recruited using the Queen’s University introductory psychology Subject Pool. Individuals were eligible to participate in the study if they met the following criteria: were over 18 years of age, were able to read and write English fluently, had normal or corrected to normal vision, and had previously viewed sexually explicit media. A total of 53 women and 22 men were included in the study, all of whom indicated exclusive or predominant sexual attractions to the other gender. This gender difference in sample size is reflective of the gender-ratio of students enrolled in the course (i.e., 2:1 ratio in favor of women). Given the results of Chivers, Bouchard, and Timmers [52] regarding patterns of genital response in exclusive versus predominantly androphilic women, we examined our data to see what effect (if any) the exclusivity of sexual attraction had on the gender difference in specificity of visual attention. Excluding the 23 women reporting predominantly androphilic sexual attractions did not influence the pattern of results in the current study; therefore, these participants were included in the final sample. Approximately half of the participants had normal vision (*n* = 37), otherwise their vision was corrected with glasses or contact lenses (*n* = 38). Table 1 includes demographic information for the sample included in the analyses separated by gender. All participants received course credit for participating in the study. All procedures were approved by the General Research Ethics Board at Queen’s University.

**Table 1:** Participant Demographic Information.

	Women	Men
	*M* (*SD*) *n* (%)	*M* (*SD*) *n* (%)
**Age**	18.6 (1.4)	20.0 (6.3)
**Relationship status**		
Single	26 (49%)	7 (32%)
Dating	27 (51%)	14 (64%)
Married	0 (0%)	1 (4.5%)
**Ethnicity**		
European	38 (72%)	14 (64%)
African	0 (0%)	1 (4.5%)
Asian	4 (7%)	4 (18%)
Hispanic	1 (2%)	1 (4.5%)
First Nations	1 (2%)	1 (4.5%)
Other	9 (17%)	1 (4.5%)
**Highest Education Completed**		
Community college (attending or completed)	0 (0%)	1 (4.5%)
University (attending/ completed bachelor’s degree)	53 (100%)	21 (95.5%)
**Employment**		
Full-time	0 (0%)	1 (4.5%)
Part-time	13 (24%)	5 (23%)
Full-time student	29 (55%)	12 (54.5%)
Unemployed	8 (15%)	3 (14%)
Other	3 (6%)	1 (4.5%)
**Hormonal Contraceptive Use**		
Yes	30 (57%)	n/a
No	23 (43%)	n/a

### Materials

#### Experimental Stimuli

Stimuli were images of nude men and women in sexually provocative poses with clearly visible aroused genitals (e.g., erect penis or engorged vulva). The images were taken from freely accessible internet sites and are available from the authors by request. Henderson [53] observed that low-level image features (e.g., luminance and complexity) have the potential to induce automatic attentional biases via bottom-up processing of these differences. To enable the semantic content of the stimuli (i.e., sexual relevance) to direct attention in a top-down rather than bottom-up manner, all images were matched for size, brightness, contrast, and colour. To do so, we first removed the background from all of the images so that each sexual target (male or female) was isolated. Using Adobe Premiere software, luminance level was assessed and manually adjusted to be consistent across images. A number of studies have shown positive correlations between compressed image file size and image file complexity, as well as positive correlations between these factors and the subjective judgment of picture complexity by participants [54, 55]. Image complexity was determined by examining the compressed image file size in JPEG format and then all images were edited to be a similar file size by adjusting the height and width of each image. The height of all pictures was set to 400 pixels and the width of all pictures varied between 250 and 300 pixels.

**Fig 1:**
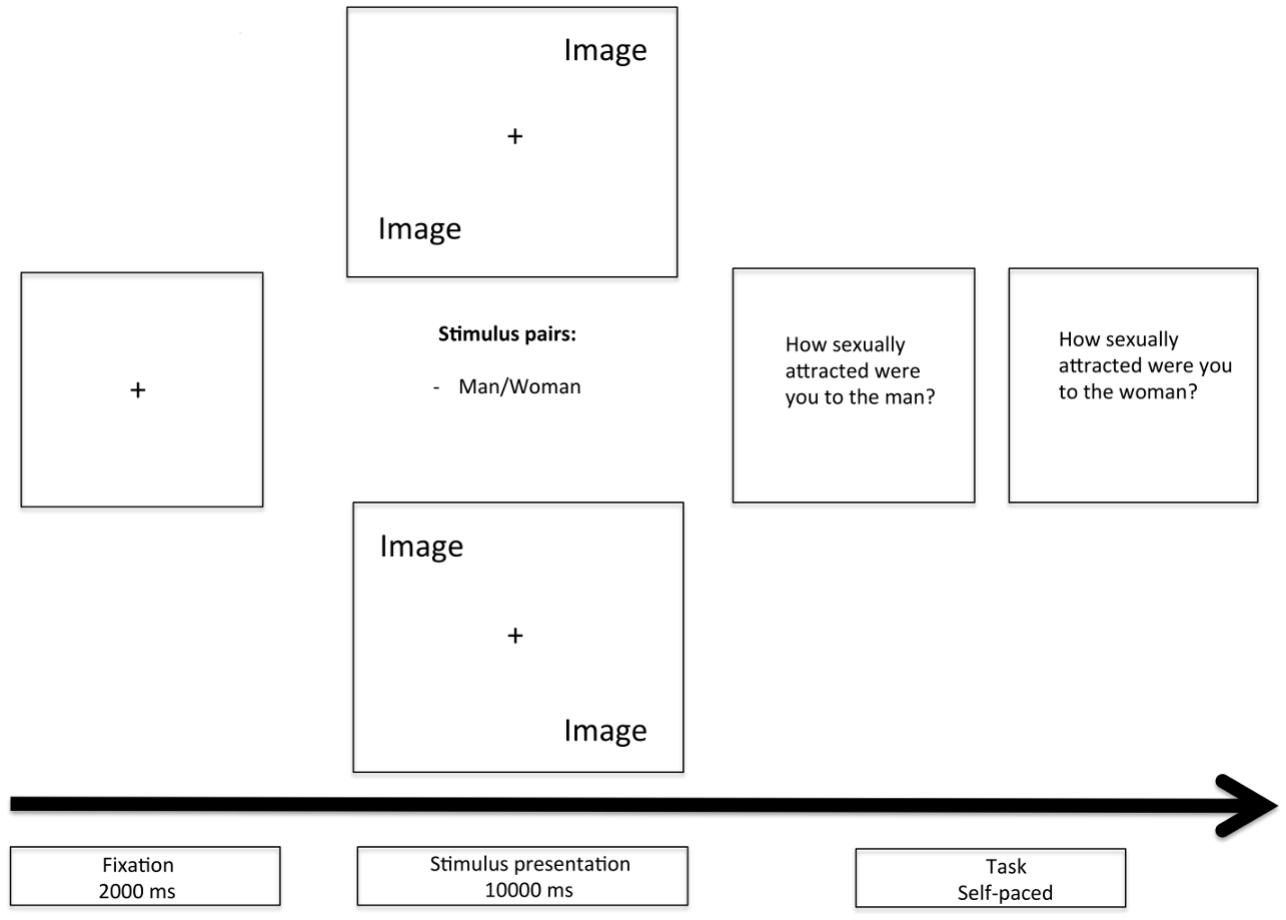
Illustration of the time sequence of a single trial.

There were 40 trials in each experimental block and three experimental blocks in total. Blocks 1 and 2 contained novel image pairs. In order to examine potential effects of familiarity on patterns of visual attention the images in block 3 were images previously used in blocks 1 and 2 in new pairings and new locations. Each trial involved two images presented simultaneously (one male target and one female target) in opposing corners of the screen (top left/bottom right or top right/bottom left), and the image location was balanced across trials, with the distance to each other and the center fixation held constant (see Fig 1). The picture pairings were matched with respect to their width and were equidistant from the center fixation point.

#### Apparatus

Eye movements were measured using a Tobii T60 eye tracker in combination with the Tobii Studio_TM_ 2.2 software. The Tobii T60 is a contact-free, remote sensor eye-tracker that measures bright and dark pupil tracking using an infrared camera. It has an automatic eye and head tracker built into a 17-inch monitor (resolution of 1280 X 1024 pixels); this tracker automatically compensates for small head movements, so it is unnecessary to immobilize the head using a chin rest. The Tobii Studio_TM_ 2.2 software works with a spatial resolution of 0.2° of visual angle, a temporal resolution of 60Hz, and a gaze position accuracy of 0.5° of visual angle. The system is compatible for use with most eyeglasses and contact lenses.

#### Post-stimulus Sexual Attraction Ratings

Following the presentation of each image pair, participants were asked to rate how sexually attracted they were to the image of the man and to the image of the woman, separately, using a 7-point scale ranging from 0 (*not at all sexually attracted*) to 3 *(moderately sexually attracted)* to 6 (*very sexually attracted*).

#### Questionnaire

Participants completed a questionnaire providing demographic information and their sexual history (see Table 1). Sexual attractions were assessed using the Kinsey scale [56, 57]. Other measures to assess sexual desire [58], sexual attitudes [59], sexual inhibition/excitation [60, 61], sexual functioning [62, 63], genital self-image [64, 65], body image [66], sexual disgust [67], homophobic attitudes [68], and anxiety [69] were administered, but these data are not examined in this paper.

### Procedure

Upon arriving to the laboratory, the experimental procedure and equipment were explained verbally, and participants provided written informed consent to participate. Participants were seated facing the monitor at eye level at a viewing distance of 60cm. During the instructions that preceded each block, participants were told that they would be asked to rate their degree of sexual attraction towards the men and women in each of the images presented, and that it was important that they look at each image carefully in order to make their judgment. This secondary task (i.e., to assess their sexual attraction to targets) may have resulted in the forced attention paradigm having a motivational component. This task differs from a free-viewing paradigm [49] where no explicit instructions or secondary tasks are included [70].

Participants were given the opportunity to rest after each of the three blocks. The first block included eight practice trials to familiarize participants with the task, followed by 40 experimental trials. During the eight practice trials, participants viewed pairs of images depicting clothed men and women. These trials followed the same procedure as the experimental trials and were intended to acclimate the participants to the procedure. The next two blocks included 40 experimental trials each (without practice trials), yielding a total of 120 experimental trials across the three blocks. The eyetracker was calibrated before each block onset (i.e., a total of 3 times throughout the experiment). The calibration followed a standard 9-point procedure that involved having the participant fixate on nine pre-determined points on the display area.

Prior to each trial, a small fixation point appeared on the center of the screen for 2 s to ensure that all participants were looking at the same point of the screen at the beginning of each trial. Following this, two images appeared and remained on the screen for 10 s. After this, two questions appeared one at a time in the same order each time. The first asked *“How sexually attracted were you to the man”* and the second asked *“How sexually attracted were you to the woman”*. Participants responded on a scale from 0 to 6 using the mouse.

### Data Analysis

#### Eye movements

Eye movement data were recorded with Tobii Studio_TM_ 2.2 software. Fixation identification was calculated using the Tobii Fixation Filter, an algorithm that identifies fixations and removes saccadic movements. The program measures the distance between neighboring gaze points and calculates the eye movement velocity for all of the eye movements sampled. The raw data points are assigned to the same fixation if the velocity remains below a set threshold, or are assigned to a new fixation when the velocity rises above this threshold (dispersal threshold of 30 pixels corresponding to 0.9° and a minimum temporal duration of 100 ms). This enables accurate calculation of fixations and does not include saccadic movements in the calculation of fixations.

In order to analyze attention towards the female and male sexual targets, we divided each stimulus display into two regions of interest (ROIs); one corresponded with the image of the male and the other corresponded with the image of the female. To eliminate any biases prior to stimulus onset, only trials where the participant’s attention was focused on the center fixation point for the 1s prior to the image pairs being presented were included in the analyses described below. To examine initial attentional biases, we calculated two dependent variables for each ROI. The ROI first fixated on within each trial (i.e., either to the image of the male or to the female) was recorded for each trial and then summed across trials [32, 40]. The latency or time taken to first fixate on a ROI was also recorded and averaged across the trials [41]. Because participants were instructed to fixate on the center fixation point prior to trial onset, first fixations were typically in the middle of the display. We characterized “first fixation” on the female or male target as the first fixation independently generated by the participant.

To examine controlled or late attentional biases towards the female and male sexual targets, we calculated total fixation duration and total fixation count for the two ROIs [32, 40]. Total fixation duration was the total amount of time spent (in seconds) in the ROI across the 10 s of presentation time, and the total fixation count was the number of fixations or times the participant’s gaze landed in the ROI. Of note, the total fixation count variable included the first fixation used in the initial attention variable number of first fixations. For each dependent variable, a 2 (Stimulus Gender: Male, Female) by 2 (Participant Gender: Man, Woman) x 3 (Trial Block: 1, 2, 3) mixed model Analysis of Variance (ANOVA) was conducted. Greenhouse Geisser corrected values are reported when the assumption of sphericity is violated. Significant interactions were further examined using Toothaker’s mixed model *t*-tests [71]. Toothaker’s mixed model *t*-tests maximize power by pooling the within- and between-subject error terms from the omnibus ANOVA. To facilitate interpretation, all effect sizes will be reported as preferred relative to nonpreferred targets.

## Results

### Self-reported Sexual Attraction Ratings

**Fig 2:**
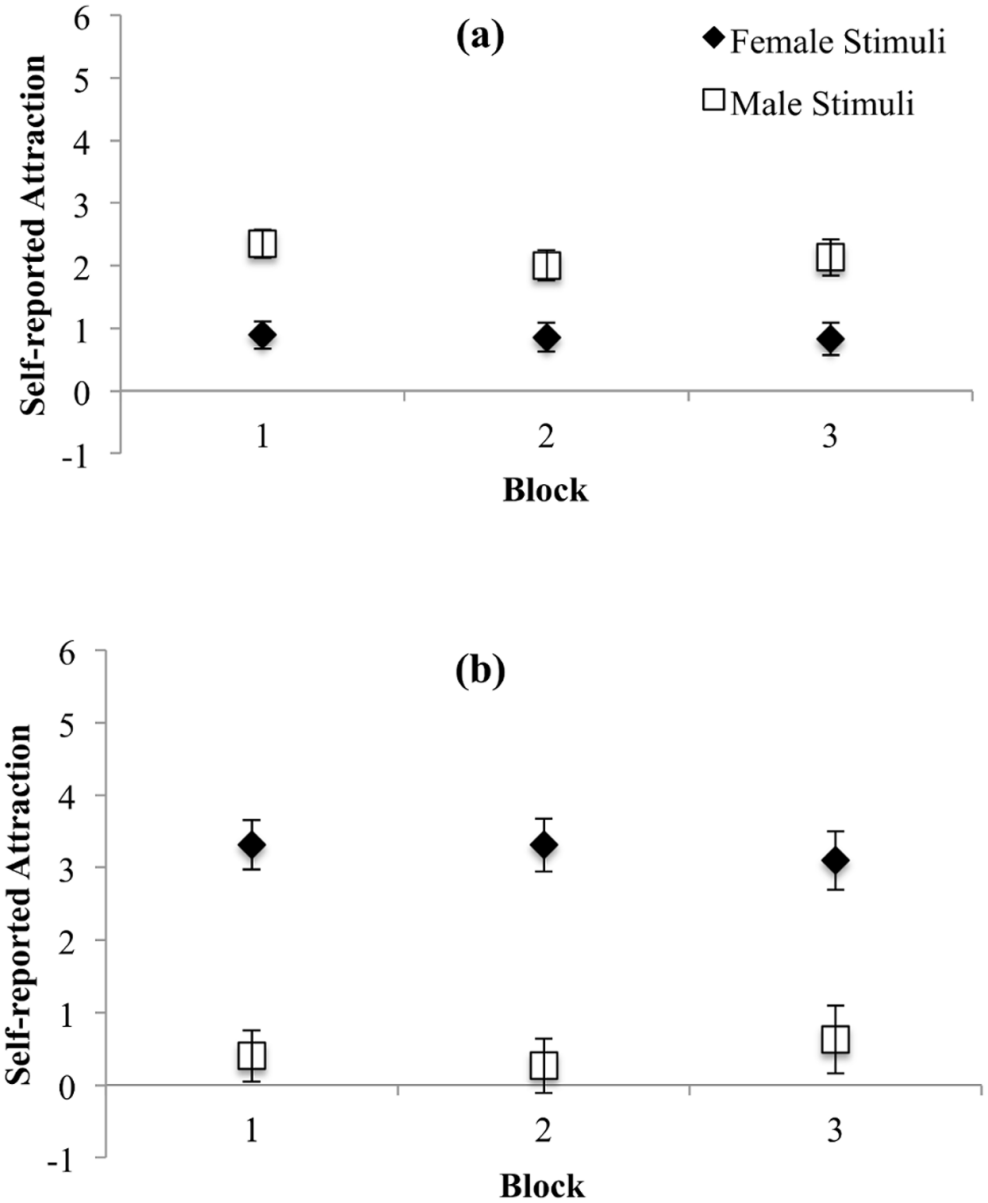
Self-reported sexual attraction ratings for female and male stimuli for women (a) and men (b). Error bars represent 95% CI.

Given that the purpose of the study was to examine attentional biases to preferred and nonpreferred stimuli, it was first important to examine whether participants did indeed report a preference for one target over another. Self-reported sexual attraction ratings for the experimental stimuli were subject to the 2 X 2 X 3 ANOVA described above. The means and standard deviations of the sexual attraction ratings as a function of ROI and Block are presented in Table 2. The ANOVA revealed a significant interaction between Stimulus Gender and Participant Gender, *F*(1, 73) = 542.85, *p* < .001, which was followed up using Toothaker’s *t*-tests for women and men separately (see Fig 2a and 2b). Women reported significantly greater sexual attraction to the male versus female targets, *t*(73) = 5.60, *p* < .001, *d* = 1.41. Men reported significantly greater sexual attraction to the female versus male targets *t*(73) = 7.77, *p* < .001, *d* = 3.53. As expected, our participants reported that they were significantly more sexually attracted to targets corresponding with their stated sexual orientation. Of note, mean attraction scores to preferred stimuli approached or fell within the “moderately sexually attracted” range, meaning that neither men nor women reported a high degree of sexual attraction towards the images presented.

**Table 2:** Average Self-reported Sexual Attraction by Block.

	Men *M* (SD)			Women *M* (SD)		
Stimulus	*B1*	*B2*	*B3*	*B1*	*B2*	*B3*
Male	0.40	0.26	0.62	2.35	2.01	2.13
	(0.51)	(0.39)	(1.19)	(0.94)	(1.01)	(1.06)
Female	3.31	3.31	3.10	0.89	0.86	0.83
	(0.82)	(0.83)	(1.13)	(0.79)	(0.87)	(0.86)

### Visual Attention

#### Initial attention: Number of first fixations and time to first fixation

Initial attentional processes were assessed using the number of first fixations and the time taken to first fixation to each of the ROIs. The means and standard deviations for these two factors as a function of ROI and Block can be seen in Tables 3 and 4.

**Table 3:** Average Number of First Fixations by Block.

	Men *M (SD)*			Women *M (SD)*		
Stimulus	*B1*	*B2*	*B3*	*B1*	*B2*	*B3*
Male	14.23	15.50	11.32	16.09	16.85	15.36
	(4.14)	(4.13)	(5.75)	(5.13)	(4.98)	(4.11)
Female	18.04	18.36	16.04	18.77	18.70	18.37
	(5.27)	(4.78)	(7.48)	(4.43)	(4.82)	(4.99)

**Table 4:** Average Time (s) to First Fixation by Block.

	Men *M (SD)*			Women *M (SD)*		
Stimulus	*B1*	*B2*	*B3*	*B1*	*B2*	*B3*
Male	1.71	1.76	1.96	1.26	1.16	1.14
	(.62)	(.82)	(1.00)	(.45)	(.38)	(.39)
Female	0.84	0.81	0.71	1.31	1.32	1.13
	(.20)	(.24)	(.28)	(.62)	(.63)	(.54)

#### Number of first fixations

**Fig 3:**
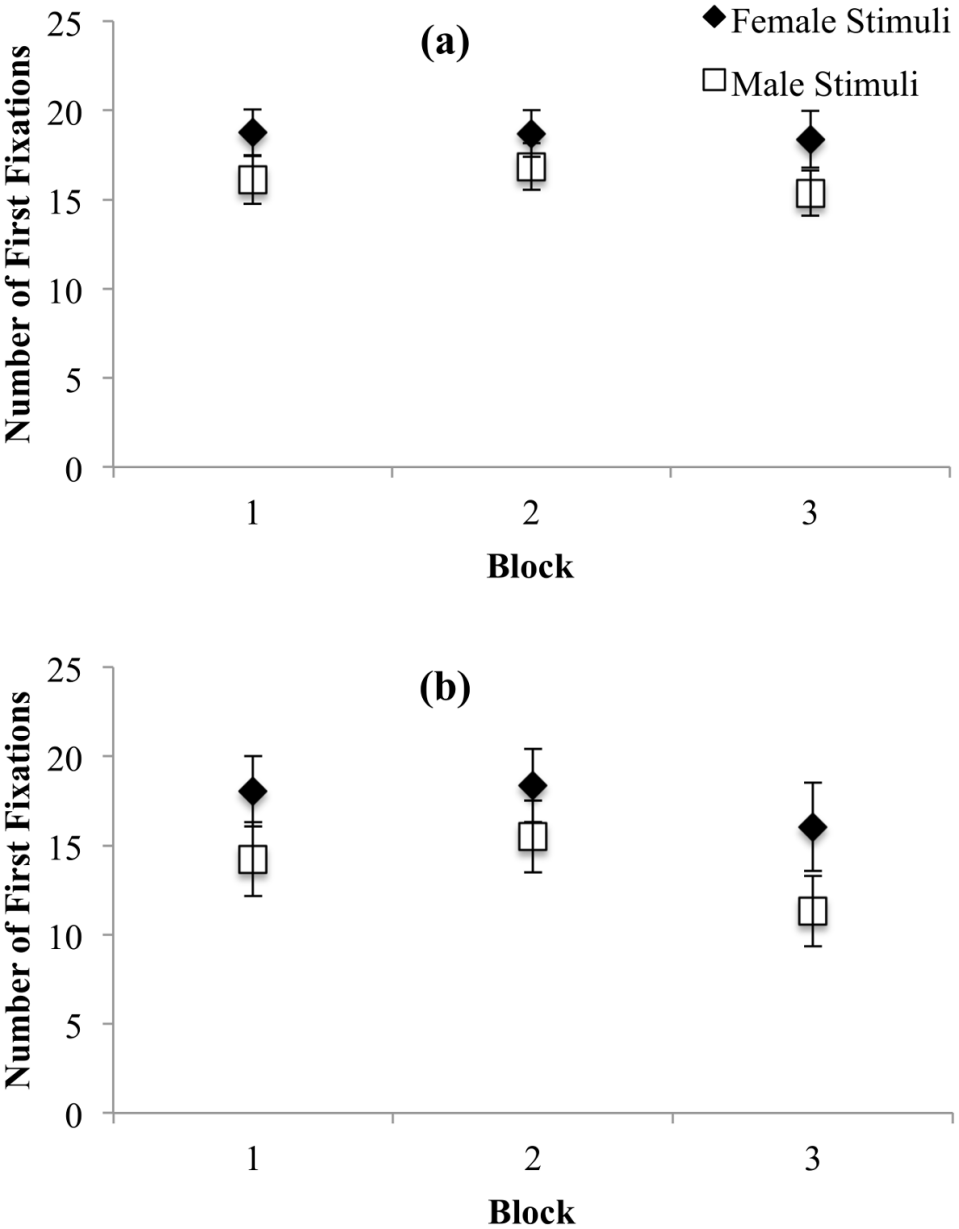
Number of first fixations towards female and male stimuli for women (a) and men (b). Error bars represent 95% CI.

Number of first fixations captures initial orienting biases, such that higher frequencies are suggestive of greater attentional capture. The results of the 2 X 2 X 3 ANOVA using the number of first fixations revealed a significant main effect of Stimulus Gender, *F*(1, 73) = 17.12, *p* < .001, η_p_^2^ = .19. For both men and women, the mean number of first fixations on female stimuli was significantly greater than the mean number of first fixations on male stimuli. There was a significant interaction between Trial Block and Participant Gender for number of first fixations, *F*(1.75, 127.45) = 4.24, *p* = .02. This interaction was examined by looking at the Trial Block effect using Toothaker’s *t*-tests separately by Participant Gender. For women, there was no significant difference in number of first fixations across blocks (all *p*s > .33 and *d*’s < .15; see Fig 3a). For men, there was a significant difference in the number of first fixations between blocks 1 and 3, *t*(73) = 1.72, *p* = .009, *d* = .46, and blocks 2 and 3, *t*(73) = 2.28, *p* = .003, *d* = .63 (see Fig 3b), such that block 3 yielded significantly fewer first fixations, on average, than the other two blocks. In sum, the number of first fixations was gender-specific for men, and, counter to prediction, significantly greater to female versus male targets in women. Stimulus familiarity did not influence patterns of gender-specificity and nonspecificity, but rather fewer first fixations were observed in men in the third trial block, which also coincided with fewer valid trials being included from block 3 for men.

#### Time to first fixation

**Fig 4:**
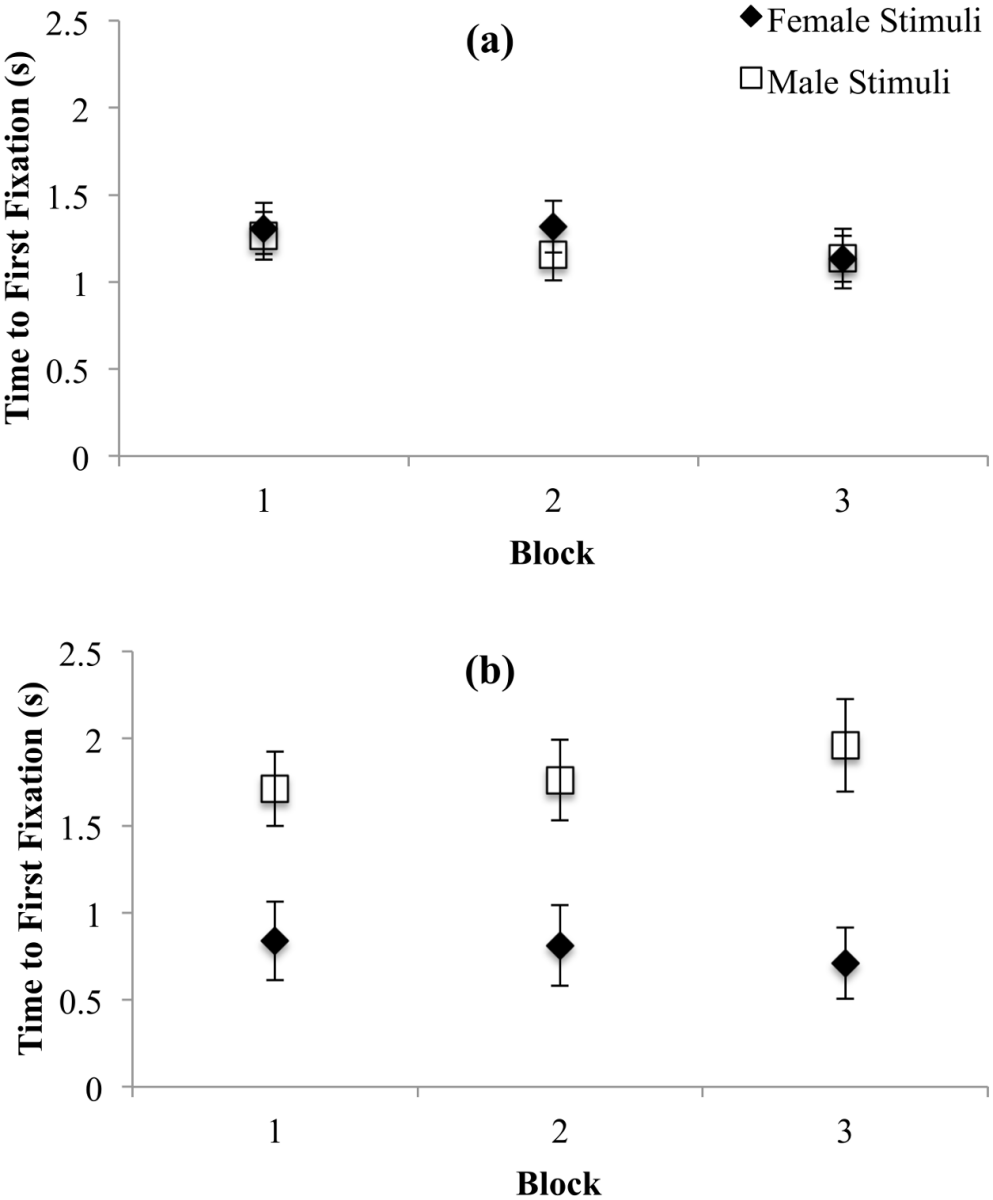
Time taken to first fixate on female and male stimuli for women (a) and men (b). Error bars represent 95% CI.

We examined time to first fixation as another dependent measure assessing initial attentional processes, whereby shorter latencies are indicative of attentional bias. The interactions between Stimulus Gender and Trial Block, *F*(1.73, 126.48) = 6.12, *p* = .004, Trial Block and Participant Gender, *F*(1.70, 124.24) = 4.80, *p* = .01, and Stimulus Gender and Participant Gender, *F*(1, 73) = 40.42, *p* < .001 were all significant. To clarify these interactions, we examined the effects of Stimulus Gender for each of the blocks separately for men and women using Toothaker’s *t*-tests. For women, time to first fixation towards male or female targets was not significantly different, across any of the blocks (all *p*s >. 16, all *d*s < .31; see Fig 4a). Women did not exhibit a bias towards male or female targets with respect to time taken to first fixation. In contrast, men oriented significantly more quickly to female than male targets in each trial block, *t*(73) = 4.99, *p* < .001, *d* = 1.91, *t*(73) = 5.43, *p* < .001, *d* = 1.58, and *t*(73) = 7.14, *p* < .001, *d* = 1.70, for blocks 1, 2, and 3, respectively (see Fig 4b). Time to first fixation was therefore gender-nonspecific for women and gender-specific for men across all three trial blocks.

#### Controlled attention: Total fixation duration and total fixation count

Controlled attentional processes were assessed using total fixation duration and total fixation count for each of the ROIs. The means and standard deviations for these two variables as a function of ROI and Block can be seen in Tables 5 and 6.

**Table 5:** Average Total Fixation Duration (s) by Block.

	Men *M (SD)*			Women *M (SD)*		
Stimulus	*B1*	*B2*	*B3*	*B1*	*B2*	*B3*
Male	2.32	1.97	1.84	4.92	4.77	4.45
	(.77)	(.81)	(.82)	(1.09)	(1.09)	(1.27)
Female	6.32	6.37	5.98	3.57	3.34	3.04
	(.84)	(.94)	(1.37)	(.94)	(.97)	(1.07)

**Table 6:** Average Total Fixation Count by Block.

	Men *M (SD)*			Women *M (SD)*		
Stimulus	*B1*	*B2*	*B3*	*B1*	*B2*	*B3*
Male	5.43	4.76	4.58	10.09	9.80	9.04
	(1.49)	(1.67)	(2.42)	(2.03)	(2.41)	(2.48)
Female	12.32	12.08	11.61	8.36	7.78	7.04
	(1.55)	(1.67)	(2.31)	(2.08)	(2.24)	(2.38)

#### Total fixation duration

**Fig 5:**
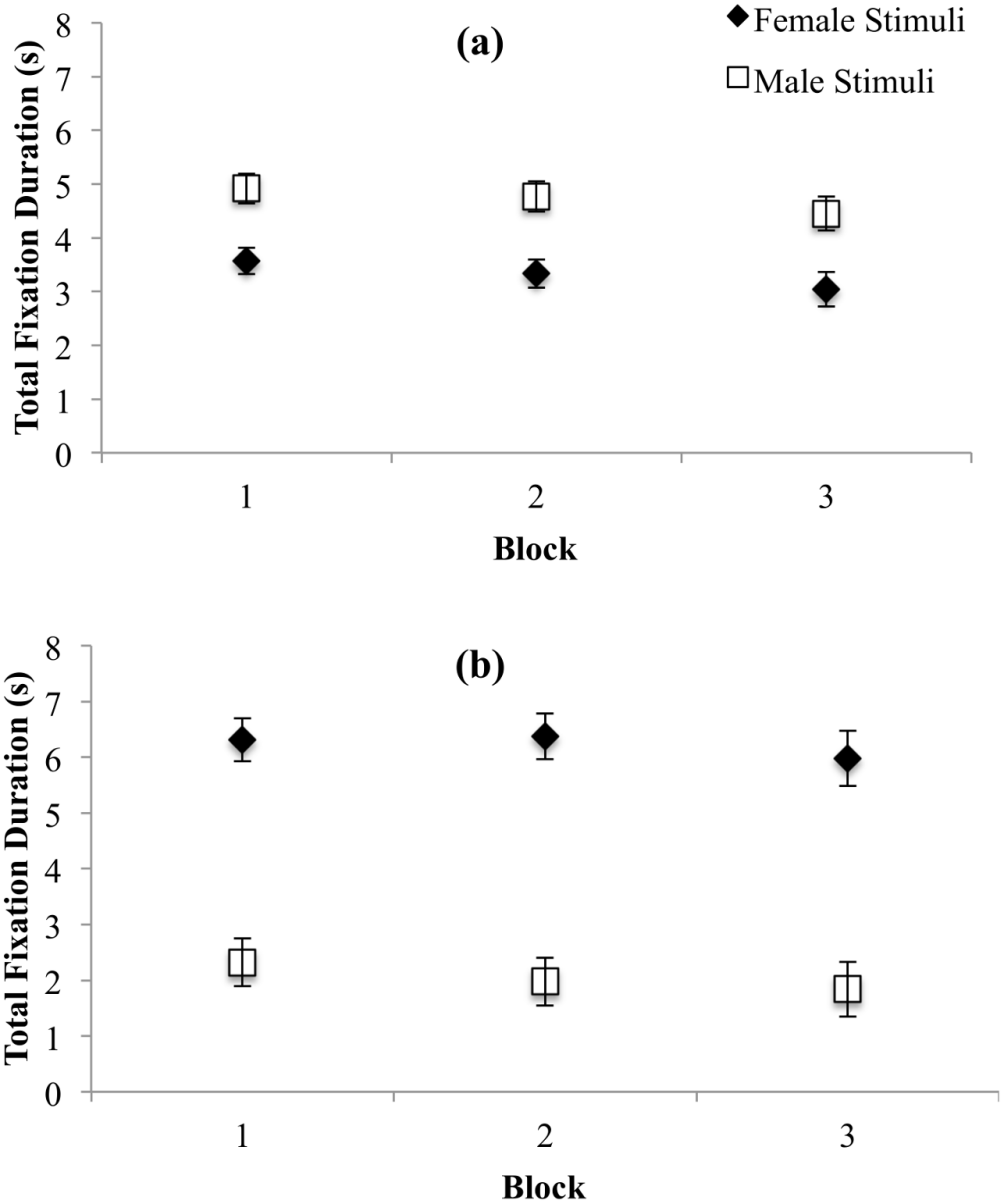
Total fixation duration on female and male stimuli for women (a) and men (b). Error bars represent 95% CI.

Total fixation duration represents the total time a participant fixated on a ROI, with longer durations indicating greater attentional engagement. The 2 X 2 X 3 ANOVA revealed a significant main effect of Trial Block on total fixation duration, *F*(1.67, 122.09) = 18.13, *p* < .001, η_p_^2^ = .20, such that total fixation durations decreased across blocks (see Table 4; all *p*s < .03). There was a significant interaction between Stimulus Gender and Participant Gender, *F*(1, 73) = 259.28, *p* < .001, which was followed up using Toothaker’s *t*-tests separately by Participant Gender. Women looked significantly longer at male targets than female targets, *t*(73) = 4.38, *p* < .001, *d* = 1.30 (see Fig 5a) and men looked significantly longer at female targets than male targets *t*(73) = 8.44, *p* < .001, *d* = 4.48 (see Fig 5b). That is, both women and men showed gender-specific patterns of total fixation duration.

#### Total fixation count

**Fig 6:**
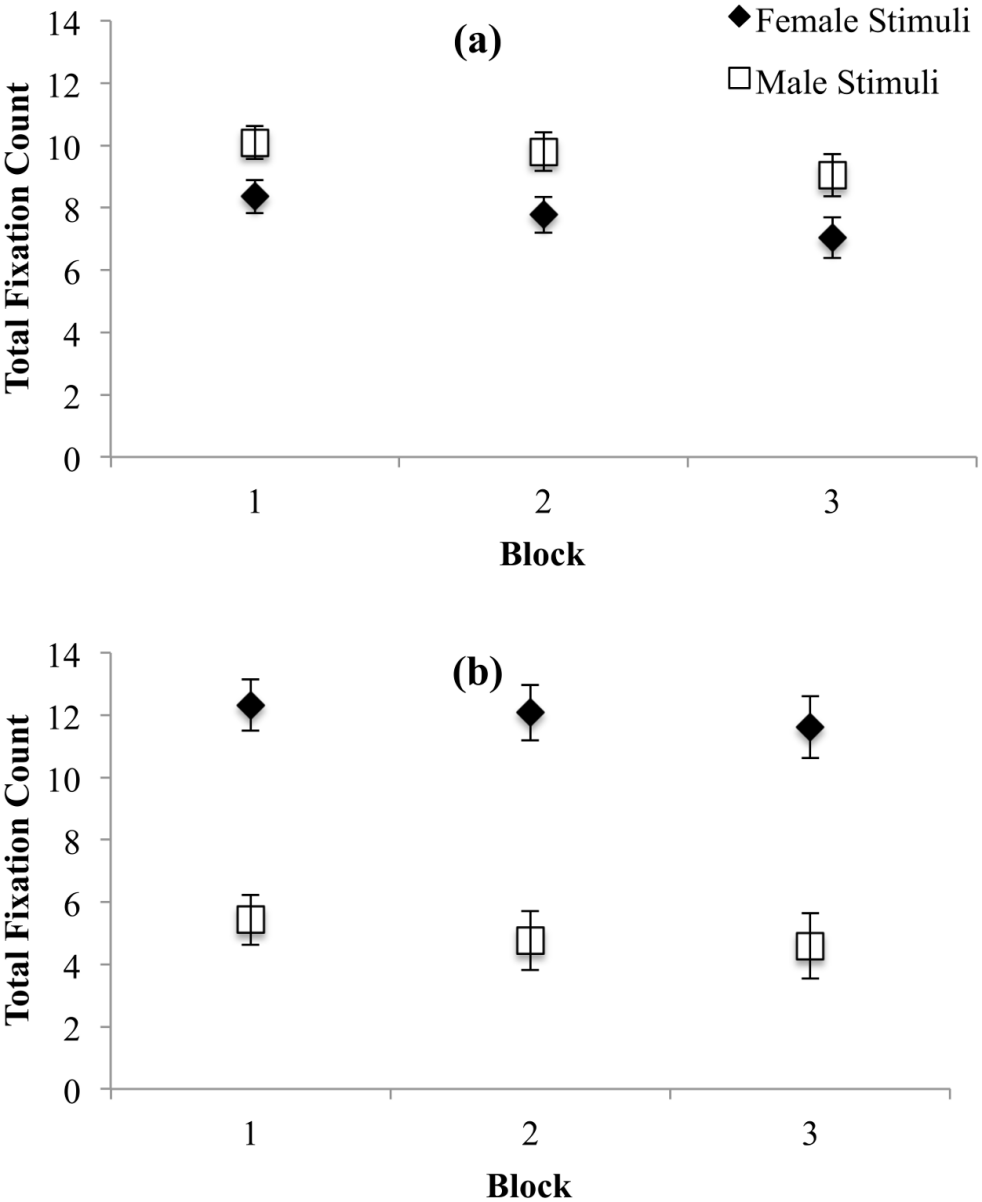
Total number of fixations on female and male stimuli for women (a) and men (b). Error bars represent 95% CI.

We examined the total number of fixations in a ROI (total fixation count), with more fixations indicating greater attentional capture and engagement. There was a significant main effect of Trial Block, *F*(1.60, 115.31) = 26.75, *p* < .001, η_p_^2^ = .27, such that total fixation count decreased across blocks (see Table 6; all *p*s < .001). The interaction between Stimulus Gender and Participant Gender was significant, *F*(1, 72) = 208.51, *p* < .001, and was followed up using Toothaker’s *t*-tests separately by Participant Gender. Women had significantly more fixations on male than female targets, *t*(73) = 2.81, *p* = .01, *d* = .85 (see Fig 6a) and men had significantly more fixations on female than male targets *t*(73) = 6.74, *p* < .001, *d* = 3.83 (see Fig 6b); that is, both women and men showed gender-specific patterns for their total fixation counts.

Previous research has reported interesting differences in gaze patterns and self-reported arousal dependent on women’s hormonal status [47, 72, 73]. We did not have any a priori hypotheses about hormonal status or hormonal contraceptive use and specificity of visual attention, but examined the effect of hormonal contraceptive use post-hoc. Hormonal contraceptive use did not significantly impact women’s patterns of results for any of the dependent measures (all *p*s > .11).

### Relationship between Visual Attention and Self-reported Attraction

We examined the relationship between gaze times and self-reported attraction ratings using within-subjects Pearson correlations and compared the strength of these correlations for men and women using Fisher’s *z*-transformation. Within-subjects correlations were used to assess the concordance of gaze time and self-reported attraction ratings across the 40 trials within each block for men and women separately. Gaze times and self-reported attraction ratings were significantly correlated for women (*r*(53) = .47, .49, and .47, respectively for blocks 1, 2, and 3) and for men (*r*(22) = .76, .75, and .68). The gender difference in the relationship between gaze and self-reported attraction was significant for Block 1, *z* = -1.74, *p* = .04; but not for Blocks 2 or 3 (*z*s = -1.55 and -1.13, *p*s = .06 and .13, respectively). Between-subjects correlations were used to examine the relationship between average gaze times and average self-reported attraction ratings to male and female stimuli across participants, to determine whether gender cues had an impact on the strength of the relationship. The strength of these correlations was then compared using asymptotic *z*-tests [74]. Across men and women, gaze times and self-reported attraction ratings were significantly positively correlated for male (*r*(75) = .71, .68, .47, all *p*s < .001 for blocks 1, 2, and 3, respectively) and female stimuli (*r*(75) = .82, .82, and .51, all *p*s < .001). The between-subjects correlation for female stimuli during Block 2 was significantly stronger than the between-subjects correlation for male stimuli (*z* = -2.21, *p* = .03). In contrast, there were no significant differences in the strength of the correlations between the female and male stimuli during Blocks 1 and 3 (*z* = -1.85, *p* = .06, *z* = -0.35, *p* = .72, respectively).

## Discussion

The current study examined the gender-specificity of initial and controlled visual attention to sexual stimuli in androphilic women and gynephilic men using a motivated-viewing paradigm. Based on predictions generated from the IPM with respect to attentional biases towards sexually-preferred and nonpreferred sexual targets, as well as gender differences in the specificity of genital and self-reported sexual arousal (e.g., [4, 5]), we expected to find a gender difference in patterns of visual attention. Specifically, we expected that gynephilic men would show an attentional bias towards preferred sexual targets, whereas androphilic women would not.

We predicted that, for men, sexually-preferred targets would be prioritized by the attentional system, such that they would attract attention more frequently and for significantly longer duration compared to nonpreferred sexual targets. Consistent with these predictions, men showed both initial (faster time to first fixation and proportion of first fixations) and controlled (total fixation duration and number of fixations) attentional biases towards female targets. Together, these findings support the hypothesis that men’s attentional system is biased towards the detection of sexually-preferred cues.

Consistent with one of our predictions, women’s patterns of initial attentional engagement were generally gender-nonspecific, such that women oriented similarly quickly to preferred targets and nonpreferred targets. Interestingly, using an alternate measure of initial attention, women oriented more often to nonpreferred targets, which is indicative of gender-specificity but in a direction incongruent with their self-stated attractions. Our finding that women’s initial attention was not biased towards their preferred gender is consistent with the findings of other researchers interested in assessing automatic processing of sexual cues in androphilic women [75, 76]. According to the IPM, the implicit processing of sexual cues is hypothesized to reflexively activate a genital response [18]. Thus, if women’s patterns of initial attention are nonspecific, this could contribute to the gender-nonspecific patterns of genital responding. Contrary to our prediction and the gender-nonspecific patterns observed in earlier studies of visual attention using images of couples [44–47], women showed a gender-specific pattern for the controlled attention variables such that preferred targets (images of males) captured attention for significantly longer durations and significantly more often than images of nonpreferred targets (females). Despite both genders exhibiting controlled attentional biases towards sexually-preferred targets, this effect was stronger among men; as noted above, we also observed a gender difference in initial attention processing. Together, these results suggest that the processing of preferred and nonpreferred sexual stimuli is gendered, and may account for differences in response patterns observed in the current study and the broader literature.

### Gendered Processing of Sexual Cues

A number of studies have examined gendered processing of sexual cues in gynephilic men and androphilic women. Janssen et al. [18] and later Spiering et al. [20, 77] observed that sexual cues were pre-attentively processed (i.e., outside of conscious awareness) in men but not women [77]. In a sample of androphilic women, Spiering et al. [77] used the paradigm from Janssen et al. and found that women were not faster at identifying sexual images after the subliminal presentation of sexual images, suggesting a lack of implicit processing of sexual content among women and a notable gender effect. In a second experiment, they observed that sexual primes facilitated sexual target recognition in women for more sexually-explicit images (i.e., clearly depicting genitals) but not for less sexually-explicit stimuli (i.e., no genitals depicted). Together, these studies suggest that the initial processing of sexual cues may differ for men and women and may be dependent on the explicitness of the sexual cues presented.

In the current study, women exhibited a gender-nonspecific pattern of initial attention, whereas men’s initial attention patterns were gender-specific. In light of the findings of Spiering et al. [77] and Janssen et al. [18], it is possible that these gender differences reflect attentional systems that have both evolved to quickly detect sexual cues, but that what constitutes a sexually-relevant cue differs for gynephilic men and androphilic women. Using implicit measures, Snowden and Gray [21] observed that both sexually-preferred and nonpreferred cues were automatically appraised as sexual for androphilic women, whereas only sexually-preferred cues were appraised as sexual for men at this initial processing stage. In our study, men preferentially attended to preferred sexual cues, thereby exhibiting an initial attentional bias whereas, for women, both preferred and nonpreferred sexual cues were detected at a similar speed. It is possible, then, that gender differences in the appraisal of these stimuli as sexual are responsible for the gendered patterns of initial attention.

Spiering et al. [77] hypothesized that other factors—which they did not explore directly (e.g., sexual motivation)—may influence implicit and explicit processing of sexual stimuli resulting in quantitative differences between men and women (i.e., differences in the magnitude of the effects). For example, Bradley et al. [49] observed that ratings of sexual disgust were negatively correlated with gaze times towards nude images of males and females in women, but not in men. Data from the current study may shed further light onto factors related to the gendered processing of sexual cues. Men and women reported that they were significantly more sexually-attracted to the images depicting preferred targets than to the images of nonpreferred targets (i.e., explicit processing); however, similar to the pattern observed for controlled attention, this effect was stronger in men. We also found that men’s and women’s fixation durations were highly correlated with their ratings of sexual attraction. Interestingly, we observed a stronger relationship between ratings of stimulus attraction and gaze times in men than in women but only for the first trial block, providing partial support for the interpretation that gender influences the relationship between attention to sexual cues and the subjective experience of sexual attraction or motivation to attend to such cues.

### Gendered Information-Processing Systems

If we assume that the processing of sexual cues is gendered in gynephilic men and androphilic women, and that this contributes to gender differences in visual attention, then the question arises as to why gendered systems would develop? Within the broader cognitive and emotion literature, there is an abundance of evidence supporting the notion that the human attentional system is prepared to detect fitness-relevant (e.g., threatening) stimuli in the environment (reviewed in [78, 79]), enabling the individual to prepare for action (e.g., to avoid or engage a potential threat). Based on ancestral gender roles and parental investment differences, Bjorklund and Kipp [80] hypothesized that gendered information-processing mechanisms may have evolved as part of sexual strategies. Given the different risks inherent in the ancestral environment, women and men may have developed gendered attentional systems that are differentially attuned to the detection of sexual cues. Men would have benefited from having an attentional system capable of quickly detecting preferred sexual targets as this would have facilitated a strategy whereby mating opportunities are maximized. In comparison, minimum reproductive costs are much higher for women (e.g., 9 months of gestation and years of child-rearing versus perhaps a few minutes of intercourse) [81]; as such, women may have developed attentional systems attuned to the detection of all sexual cues, in order to approach or avoid sexual encounters based on an evaluation of mate quality. In addition, there is substantial evidence to suggest that, within the ancestral environment, women were threatened by unwanted sexual experiences [82]; therefore, women’s attentional system may have evolved the capacity to rapidly detect sexual cues (preferred or nonpreferred) in order to avoid these threats or to physically prepare the body for sexual activity [83].

Researchers have postulated that women’s genital responses are reflexively activated by sexual cues—provided the stimuli contain a frankly sexual cue—irrespective of whether the cues are preferred or nonpreferred, in order to protect the vaginal lumen from potential injury [4, 5, 11, 83–86]. If automatic processing of sexual stimuli involves the initial detection of sexually-relevant features in order to trigger a sexual response, then it is possible that our finding of nonspecific initial attention in androphilic women is responsible for the activation of nonspecific genital response patterns. Controlled attention, on the other hand, involves the elaborative processing of sexual cues, and was strongly related to reported attraction in both genders. Controlled attention patterns were gender-specific for gynephilic men and androphilic women, and as such may be more strongly related to patterns of self-reported arousal or sexual orientation.

### Future Directions

The finding that women’s controlled attention was gender-specific differs from the patterns of nonspecificity of visual attention and genital response that have been reported in the literature [4, 5, 8, 11, 13–17, 44–48]. We hypothesize that differences in the duration and types of stimuli used in these studies may contribute to these seemingly discrepant findings. We presented two sexually-explicit single-target images simultaneously for 10 seconds, which is considerably shorter duration than the 90 to 120 seconds of coupled or individual sexual stimuli typically used to assess specificity of sexual response and the coupled stimuli used in studies of visual attention. In the current study, we observed gender-nonspecific patterns of initial attention in androphilic women, whereas specificity of attention emerged during the presentation of the stimuli. It is possible that, if stimuli were presented for longer durations, nonspecific controlled attention patterns in androphilic women would emerge. An alternate, but not necessarily mutually exclusive hypothesis is that initial attentional processes trigger an automatic genital response that is not inhibited during the explicit processing of lengthier stimuli, which may involve gender-specific controlled attention in androphilic women.

The stimuli used in the current study also differed from those typically used in studies of visual attention and sexual arousal, such that we presented static nude images of individuals in sexually aroused states, rather than images or dynamic stimuli of individuals or couples engaging in sexual activities. These stimulus features (e.g., movement or contextual cues) in combination with, or independent of, stimulus length may also affect the explicit processing of sexual stimuli and subsequent attention and/or genital responding. Spape et al. [87] recently observed specificity of genital response among androphilic women and gynephilic men when static images of aroused genitals were used as stimuli. It is possible that stimuli with minimal contextual cues may influence the explicit processing of sexual stimuli in such a way that features of sexually-preferred targets are elaborated upon more fully than features of nonpreferred sexual targets. Using dynamic videos as stimuli, Tsujimura et al. [48] observed a gender-nonspecific pattern of controlled visual attention in women and a gender-specific pattern in men, suggesting that contextual cues impact the explicit processing of sexual stimuli differently for men and women. Future studies should assess visual attention and physiological arousal concurrently using static and dynamic stimuli in order to further test the IPM and understand the potential attentional mechanisms contributing to patterns of gender specificity and nonspecificity that have been observed in the literature.

In the current study we sought to examine gender differences and similarities in attentional biases to sexual cues in a sample of gynephilic men and androphilic women. Although we discuss our findings in terms of a gender difference and hypothesize gendered processing of sexual cues, we recognize that comprehensive effects of gender are best examined using a more diverse sample of individuals rather than a sample limited to gynephilic men and androphilic women. Specifically, the inclusion of men and women of diverse sexual attractions, as well as cis- and transgender individuals, would allow for a more thorough examination of gender effects on response patterns. We also note that our recruitment method was limited such that it resulted in uneven samples of men and women, which may have impacted our ability to make gender comparisons as the populations sampled may have differed from one another. Future research should include a more diverse sample, as well as more comparable numbers of men and women in order to draw firm conclusions about gender and sexual attractions (for example the samples in [75, 76]).

## Conclusions

The current study is the first to examine initial and controlled attention patterns in a sample of androphilic women and gynephilic men. Initial and controlled attentional processing in gynephilic men serves to detect and attend to preferred sexual cues only (i.e., gender-specific responding), whereas initial and controlled attentional processing in androphilic women yield patterns of gender-nonspecificity and specificity, respectively. The observed patterns of initial visual attention correspond nicely with genital response patterns observed in other studies and provide preliminary evidence that attention may be one mechanism driving these effects. The strong relationship between controlled attention and sexual attraction supports the use of the forced attention paradigm for assessing sexual preference in both androphilic women and gynephilic men.
