# Correction: Gender-Specificity of Initial and Controlled Visual Attention to Sexual Stimuli in Androphilic Women and Gynephilic Men

**DOI:** 10.1371/journal.pone.0155651

**Published:** 2016-05-10

**Authors:** The PLOS ONE Staff

In the Funding section, the name of the “Natural Sciences and Engineering Research Council of Canada Discover Grant” is listed incorrectly. The correct funder name is: Natural Sciences and Engineering Research Council of Canada Discovery Grant.
